# The impact of provider restrictions on abortion-related outcomes: a synthesis of legal and health evidence

**DOI:** 10.1186/s12978-022-01405-x

**Published:** 2022-04-18

**Authors:** Fiona de Londras, Amanda Cleeve, Maria I. Rodriguez, Alana Farrell, Magdalena Furgalska, Antonella F. Lavelanet

**Affiliations:** 1 Birmingham Law School, University of Birmingham, B15 2TT Birmingham, UK; 2 Women’s and Children’s Health, Karolinska Institute, Stockholm, Sweden; 3 Department of Obstetrics and Gynecology, Oregon Health and Science University, Portland, OR USA; 4 York Law School, University of York, York, UK; 5 UNDP-UNFPA-UNICEF-WHO-World Bank Special Programme of Research, Development and Research Training in Human Reproduction (HRP), Department of Sexual and Reproductive Health and Research, World Health Organization, Geneva, Switzerland

**Keywords:** Abortion, Provider restrictions, Reproductive health, Law and policy, Human rights, Abortion law and policy

## Abstract

Many components of abortion care in early pregnancy can safely be provided on an outpatient basis by mid-level providers or by pregnant people themselves. Yet, some states impose non-evidence-based provider restrictions, understood as legal or regulatory restrictions on who may provide or manage all or some aspects of abortion care. These restrictions are inconsistent with the World Health Organization’s support for the optimization of the roles of various health workers, and do not usually reflect evidence-based determinations of who can provide abortion. As a matter of international human rights law, states should ensure that the regulation of abortion is evidence-based and proportionate, and disproportionate impacts must be remedied. Furthermore, states are obliged take steps to ensure women do not have to undergo unsafe abortion, to reduce maternal morbidity and mortality, and to effectively protect women and girls from the physical and mental risks associated with unsafe abortion. States must revise their laws to ensure this. Where laws restrict those with the training and competence to provide from participating in abortion care, they are *prima facie* arbitrary and disproportionate and thus in need of reform. This review, developed by experts in reproductive health, law, policy, and human rights, examined the impact of provider restrictions on people seeking abortion, and medical professionals. The evidence from this review suggests that provider restrictions have negative implications for access to quality abortion, contributing *inter alia* to delays and recourse to unsafe abortion. A human rights-based approach to abortion regulation would require the removal of overly restrictive provider restrictions. The review provides evidence that speaks to possible routes for regulatory reform by expanding the health workforce involved in abortion-related care, as well as expanding health workers' roles, both of which could improve timely access to first trimester surgical and medical abortion, reduce costs, save time, and reduce the need for travel.

**Supplementary Information:**

The online version contains supplementary material available at 10.1186/s12978-022-01405-x.

## Plain language summary

This review identifies evidence of the impacts of provider restrictions on people seeking to access abortion and on abortion providers. It pursues a methodology designed to ensure the full integration of public health and human rights standards developed by the research team and published elsewhere. The evidence from this review points clearly to provider restrictions having negative implications for health outcomes, health systems, and human rights. This is especially important as international guidance provided by the WHO indicates best practice in provision and management of abortion and shows clearly that undue provider restrictions are not justified by reference to the nature and complexity of abortion.

**Supplementary Information:**

The online version contains supplementary material available at 10.1186/s12978-022-01405-x.

## Introduction

Many components of abortion care in the first trimester can safely be provided on an outpatient basis by mid-level providers or by pregnant people themselves. Yet, some states impose provider restrictions [1], understood as legal or regulatory restrictions on who may provide all or some aspects of abortion care. These restrictions are arbitrary: they are inconsistent with the World Health Organization’s (WHO) support for the optimization of the roles of various health workers [2, 3], and do not usually reflect evidence-based determinations of who can provide abortion.

Expanding the role of health workers involved in abortion care can increase the availability and accessibility of quality abortion care and lead to the better enjoyment of the internationally protected right to sexual and reproductive health ([4], para 12). Since its foundation, the WHO has recognized that “[t]he enjoyment of the highest attainable standard of health is one of the fundamental rights of every human being” [5], and human rights are integrated into its work.

International human rights law increasingly reflects the proposition that the availability and accessibility of abortion—rather than “mere” legality—is of critical importance ([6], para 8) to ensuring the effective realization of a wide range of reproductive rights, including the rights to privacy, life, security of person, and freedom from torture, cruel, inhuman and degrading treatment or punishment. As a matter of international human rights law, states should ensure that the regulation of abortion is evidence-based (i.e. not arbitrary) and proportionate (i.e. provided for by law, necessary for and rationally connected to the achievement of a legitimate objective that is pursued through the regulation, and minimally intrusive) ([7], para 18). Disproportionate impacts must be remedied ([8], para 8). Where laws restrict those with the training and competence to provide from participating in abortion care, they are *prima facie* arbitrary and disproportionate.

The aim of this review is to address knowledge gaps related to the health and non-health outcomes of provider restrictions through the effective synthesis of both human rights standards and evidence from existing studies using a methodology for integrating human rights in guideline development that has been described elsewhere [9]. This methodology is well-suited to interventions that are complex and can have multiple components interacting synergistically or dissynergistically, may be non-linear in their effects, and are often context dependent [10]. Such complex interventions often interact with one another, such that outcomes related to one individual or community may be dependent on others, and may be impacted positively or negatively by the people, institutions and resources that are arranged together within the larger system in which they are implemented [10]. This review is one of seven such reviews that were carried out as part of developing the evidence base for the WHO’s new consolidated *Abortion Care Guideline* (2022) [11].

Throughout this review we use the terms women, girls, pregnant women [and girls], pregnant people, and people interchangeably to include all those with the capacity for pregnancy.

## Methods

### Identification of manuscripts and data extraction

This review examined the impact of provider restrictions on two populations (i) people seeking abortion, and (ii) medical professionals. The search strategy was developed together with experts working in the fields of law, policy and human rights. It included the key words ‘abortion AND provider restriction’, ‘abortion AND provider regulation’, ‘abortion AND healthcare providers’. The search strategy is included in Additional file 1: Appendix S1. We searched the databases PubMed, HeinOnline, and JStor and the search engine Google Scholar. We looked for new evidence that was not included in the last update of the WHO guidelines: we therefore limited our search from 2010 through July 2021. Only manuscripts that undertook original data collection or analysis were included; we included quantitative studies (comparative and non-comparative), qualitative and mixed-methods studies, reports, PhD theses, and economic or legal analyses. Recognising that country experiences of provider restrictions may provide evidence about their impacts on abortion-related outcomes, no geographic limitations were imposed.

The full review team comprised of 6 members (MF, AF, FdL, AC, MR and AL). Two reviewers (MF and AF) conducted an initial screening of the literature. Titles and abstracts were first screened for eligibility using the Covidence® tool [12]; full texts were then reviewed. We restricted our analysis to English language outputs only. A third reviewer (FdL) confirmed that these manuscripts met inclusion criteria. Two reviewers (FdL and AC) extracted data. Any discrepancies were reviewed and discussed with two additional reviewers (AL and MR). The review team resolved discrepancies through consensus.

Our outcomes of interest included both health and non-health outcomes that, based on a preliminary assessment of the literature [13], could be linked to the effects of the provider regulation intervention. Our a priori outcomes included delayed abortion, opportunity costs, self-managed abortion, workload implications, system costs, perceived imposition on personal ethics or conscience, perceived impact on relationship with patient, referral to another provider, unlawful abortion, continuation of pregnancy, or stigmatization. A preliminary human rights analysis was also undertaken, drawing on the international human rights corpus on reproductive rights [9].

In order to fully understand the implications of the findings for abortion law and policy, we applied human rights standards to the data extracted from these manuscripts. The applicable standards from human rights law were drawn from a careful review of the corpus of international human rights law in accordance with the approach outlined elsewhere [9]. They thus exclude regional and national human rights laws. The applicable standards were considered together with the evidence from the included manuscripts in order to identify, (a) which human rights standards are engaged by provider restrictions, (b) whether this evidence suggests that provider restrictions have positive or negative effects on the enjoyment of rights, and (c) where no data is identified from the manuscripts against outcomes of interest, whether human rights law provides evidence that can further elucidate the impacts and effects of provider restrictions. This is summarized in Tables 2 and 3 below.

### Analysis

We matched data from included studies to the outcomes of interest and presented this in evidence tables. In these tables, the association of each finding on the outcome was presented, as well as an overall conclusion of the identified findings across the body of evidence. We then applied human rights standards to these outcomes to develop an understanding of the effects of provider restrictions that combines the evidence from human rights law (i.e. the applicable human rights standards) and the included studies. To summarize the effect of the intervention, across all study designs, we used and applied a visual representation of effect direction. The direction of the evidence was illustrated by a symbol which indicated whether, in relation to that particular outcome, the evidence extracted from a study suggested an increase (▲), decrease (⊽), or no change in the outcome (○). The symbol did not indicate the magnitude of the effect [9].

## Results

**Fig. 1:**
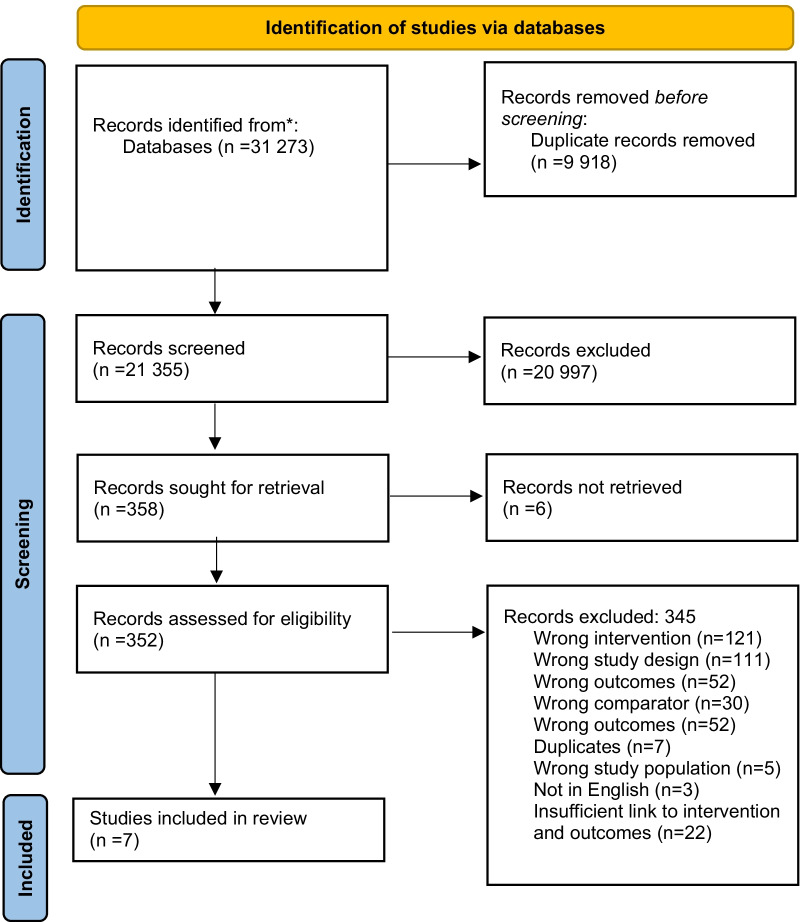
PRISMA Flow diagram

The search generated 27,480 citations after duplicates were removed. We screened the titles and abstracts and conducted a full text screening of 389 manuscripts. We excluded those manuscripts that did not have a clear connection with the intervention and our pre-defined outcomes, resulting in 9 manuscripts being included in the final analysis (Fig. 1. Prisma flow diagram).

Manuscripts described data from four countries: Australia [14, 15], Ethiopia [16, 17], Nepal [18] and the United States [15, 19–22]. The characteristics of included manuscripts are presented in Table 1. The included studies contained information relevant for the outcomes: delayed abortion [19–21]; opportunity costs [15, 16, 18–22]; self-managed abortion [16]; system costs [2, 14, 19, 21, 22]; workload implications [14, 17–20, 22]; perceived imposition on personal ethics or conscience [20]; and perceived impact on relationship with patient [20]. No evidence was identified linking the intervention to the outcomes: referral to another provider; unlawful abortion; continuation of pregnancy; or stigmatization.

**Table 1:** Characteristics of included studies

Author/year	Country	Methods	Participants	Applicable Provider Restriction
Afework 2015	Ethiopia	Individual interviews at three private health facilities	Women seeking abortion services n = 38, health extension workers n = 9, healthcare providers n = 7	No provider restrictions in legislation; determined by ministerial guidelines. Elements of CAC may be provided by gynecologists, General Practitioners (GPs), health officers, IESO and nurse midwives, nurses and health extension workers
Andersen 2016	Nepal	Program evaluation including baseline and post-training evaluation using provider progress reports and interviews	N = 290 primary level facilities providing medical abortion. Interviews with 98 healthcare providers	Provision of medical abortion expanded from physicians and staff nurses to auxiliary nurse-midwives already certified as skilled birth attendants
Battistelli 2018	United States	Individual interviews	Administrators n = 20 whose 5 organizations trained and employed healthcare providers in surgical abortion	Provision of first-trimester aspiration abortions extended to nurse practitioners, certified nurse-midwives, and physician assistants
Bridgman-Packer 2018	Ethiopia	Case study including a desk review and individual interviews	Healthcare providers n = 3, government officials n = 1, NGO staff n = 4	No provider restrictions in legislation; determined by ministerial guidelines. Elements of CAC may be provided by gynecologists, GPs, health officers, IESO and nurse midwives, nurses and health extension workers
De Moel-Mandel 2019	Australia (Victoria)	Delphi process	Healthcare providers n = 17, experts involved with or interested in medical abortion provision Of 24 participants 17 completed 3 rounds	Prescription for medication abortion only permitted by physicians who have completed a particular online training program
Grossman 2015	United States and Australia	Legal commentary	N/A	Prescription for mifepristone limited to certified physicians (GPs who have completed online training, or obstetrician-gynecologists exempt from the online training)
Mercier 2015	North Carolina, United States	In-depth individual interviews	Abortion providers (17 physicians, 9 nurses, 1 physician assistant, 1 counsellor and 3 clinic administrators)	Women´s Right to Know Act (WRTK) which mandates that counselling is conducted by licensed medical professionals
Rasmussen 2021	Illinois, United States	In-depth interviews	19 primary care clinicians and administrators (7 family physicians, 3 nurse practitioners, 4 certified nurse midwives, 5 administrators)	Food and Drug Administration (FDA) Risk Evaluation and Mitigation Strategy (REMS) for mifespristone, which requires providers to be certified with the manufacturer and complete a Prescriber Agreement Form, patients to sign a Patient Agreement Form, and medication to be dispensed only in a clinical, medical office or hospital
Srinivasulu 2021	United States	Online qualitative surveys	113 primary care clinicians (67 family physicians, 17 midwives, 12 nurse practitioners or physician assistants, 9 other physician types, 8 no response)	Food and Drug Administration (FDA) Risk Evaluation and Mitigation Strategy (REMS) for mifespristone, which requires providers to be certified with the manufacturer and complete a Prescriber Agreement Form, patients to sign a Patient Agreement Form, and medication to be dispensed only in a clinical, medical office or hospital

### Impact of the intervention on abortion seekers

A summary of the impacts of the intervention on abortion seekers and the application to human rights are presented in Table 2. Evidence identified per study and outcome are presented in Additional file 2: Tables S1 and S2. The evidence from three studies [19–21] suggests that provider restrictions contribute to delayed abortion by demonstrating how expansion of health workers’ roles improve timely access to care [19] and by showing how requiring a specific provider, who must also undertake mandated scripted counselling, imposes logistical and administrative burdens which in turn may lead to delayed abortion [20, 21]. Provider restrictions that do not reflect the evidence on who has the necessary skills to provide quality abortion [11] and which produce or contribute to delays in accessing abortion are likely arbitrary and disproportionately interfere with the human rights of pregnant people. In particular they suggest non-compliance with states’ obligation to respect, protect and fulfil the right to life and the right to health, and particularly the obligation to take steps to reduce maternal mortality and morbidity ([6], para 8; [23], paras 6, 9, 24, 30–33), and to ensure that, where it is lawful, abortion is safe and accessible ([6], para 8).

**Table 2:** Impact of PR on the abortion seeker (A + B + C)

Outcome	Overall conclusion of evidence (A)	Application of HR standards (B)	Conclusion evidence + HR (C)
Delayed abortion	Overall, evidence from three studies suggests that provider restrictions may result in delayed abortions One study indirectly examines provider restrictions on delayed abortion by demonstrating how expansion of health worker roles (and thereby reducing provider restrictions) improve timely access to first trimester surgical and medical abortion Evidence from two studies suggests that government mandated abortion counselling increases the administrative and logistical burdens for providers and women, and may increase abortion delays	Provider restrictions engage states’ obligation to respect, protect and fulfil rights to life and health (by taking steps to reduce maternal mortality and morbidity including addressing unsafe abortion, and by ensuring abortion regulation is evidence-based and proportionate)	Delayed access to abortion care can have negative impacts on the right to life, health, and to physical and mental integrity. Provider restrictions that are not justified by evidence (e.g., of competence, effectiveness, acceptability) interfere disproportionately with rights
Continuation of pregnancy	No evidence identified	Provider restrictions engage states’ obligations to protect, respect and fulfil the right to health (by ensuring abortion regulation is evidence-based and proportionate) and the right to decide on the number and spacing of children. They may also result in violations of the state’s obligation to ensure abortion is available where the life and health of the pregnant person is at risk, or where carrying a pregnancy to term would cause her substantial pain or suffering, including where the pregnancy is the result of rape or incest or where the pregnancy is not viable	If provider restrictions not based in evidence result in undesired continuation of pregnancy, this has negative impacts for rights to health, physical and mental integrity, privacy, and potentially the right to be free from torture, inhuman and degrading treatment or punishment
Opportunity costs	Overall evidence from seven studies suggests that provider restrictions increase opportunity costs for abortion seekers Provider restrictions may be linked to opportunity costs such as increased financial costs, need for travel, waiting times, additional clinic contacts, emotional distress, and undesired surgical interventions	Provider restrictions engage states’ obligation to respect, protect and fulfil rights to life and health (by ensuring where it is lawful, abortion is safe and accessible, by ensuring abortion regulation is evidence-based and proportionate), and the right to equality and non-discrimination	Provider restrictions that are not justified by evidence (e.g., of competence, effectiveness, acceptability) interfere disproportionately with rights to health and to physical and mental integrity. Provider restrictions can particularly affect marginalized women and women in rural areas with negative implications for their right to equality and non-discrimination in access to healthcare
Unlawful abortion	No evidence identified	Provider restrictions engage states’ obligation to respect, protect and fulfil rights to life and health (by taking steps to reduce maternal mortality and morbidity including addressing unsafe abortion, and by protecting people seeking abortion)	If provider restrictions not based in evidence result in inaccessibility of lawful abortion and recourse to unlawful abortion, which may be unsafe, this has negative impacts for rights to health, physical and mental integrity, and privacy
SMA	Overall evidence from one study suggests that provider restrictions, when they limit access to care, may be linked to unsafe self-managed abortion	Provider restrictions engage states’ obligation to respect, protect and fulfil rights to life and health (by taking steps to reduce maternal mortality and morbidity including addressing unsafe abortion, by ensuring abortion regulation is evidence-based and proportionate, and by protecting people seeking abortion)	Where provider restrictions lead abortion seekers to self-manage their abortions outside the formal health system, and where such self-managed abortion is unsafe, the provider restrictions have negative implications for rights
Referral to another provider	No evidence identified	N/A	Where provider restrictions preclude a healthcare provider from providing abortion care, immediate referral to a qualified and willing provider may ensure lawful abortion is safe and accessible for the abortion seeker

Findings from seven studies [15, 16, 18–22] suggest that provider restrictions increase opportunity costs including increased financial cost, travel time and associated costs, waiting times, additional clinic contacts, emotional distress for abortion seekers, and undesired surgical interventions. These opportunity costs again point to potential incompatibility with human rights, including the right to equality and non-discrimination in sexual and reproductive health. Four studies [15, 16, 18, 19] provide evidence on the positive effects of expanded health worker roles, which include reduced costs, need for travel and waiting times, and improved access to abortion.

One study [16] found that provider restrictions may limit access to care and contribute to unsafe self-managed abortions. International human rights law includes an obligation on states to take steps to reduce maternal mortality and morbidity and to protect people seeking abortion including from the physical and mental risks associated with unsafe abortion ([6], para 8). While self-managed abortion is not inevitably unsafe, the state is obliged to ensure that its regulatory choices—including provider restrictions—do not force women to resort to unsafe abortion and, if necessary, to review, reform and liberalize its laws to achieve this ([8], para 28). Considered alongside human rights law, these studies thus suggest that provider restrictions that do not reflect the evidence on who has the necessary skills to provide quality abortion [11] result in disproportionate interferences with the rights of people seeking abortion.

### Impact of provider restrictions on health professionals

A summary of the impacts of the intervention on health professionals and the application of human rights are presented in Table 3. Evidence identified per study and outcome are presented in Additional file 2: Tables S1 and S2. Evidence from six studies [14, 17–20, 22]; suggests that provider restrictions have workload implications for healthcare professionals. These include issues such as sustainability of staffing, logistical and financial costs, organizational changes, increased workload, and stress experienced by medical professionals. The process of expanding health worker roles involves challenging the traditional division of labour [14, 17] and could require changes to staffing and logistics and increased costs in the short term [19]. Workload implications of this kind may result in persons or facilities not providing abortion care or arranging care only in very constrained ways (e.g., one day a week or similar) so that, in reality, access to abortion is obstructed by provider restrictions. States’ obligation to respect, protect and fulfil the right to the highest attainable standard of physical and mental health includes an obligation to ensure sexual and reproductive health care is available, accessible, acceptable and of good quality ([4], paras 8, 12). These studies suggest that the impact of provider restrictions that do not reflect the evidence on who has the necessary skills to provide quality abortion [11] may be to make abortion less available and accessible, and thus be inconsistent with human rights.

**Table 3:** Impact of PR on the abortion provider (A + B + C)

Outcome	Overall conclusion of evidence (A)	Application of HR standards (B)	Conclusion evidence + HR (C)
Workload implications	Overall evidence from six studies suggests that provider restrictions have workload implications Four of the five studies examined this indirectly, by demonstrating the benefit in task sharing abortion care with health workers who are not physicians. One study directly examined workload implications from provider restrictions with mandated counselling All studies reported that provider restrictions may be linked with a range of workload implications including issues surrounding sustainability of staffing, logistical and financial costs, organizational changes, increased workload and stress among providers	Provider restrictions engage states’ obligation to respect, protect and fulfil rights to life and health (by ensuring abortion regulation is evidence-based and proportionate, and by protecting healthcare professionals providing abortion care)	Workload implications arising from provider restrictions that are not justified by evidence (e.g., of competence, effectiveness, acceptability) may place significant burdens on healthcare professionals providing abortion care, with negative implications for both their rights and the rights of persons seeking to access abortion
System costs	Overall, evidence from five papers suggests that provider restrictions contribute to increased system costs Provider restrictions contribute to costs at the individual, provider and systems level. For individuals, these costs are typically associated with increased time in obtaining care. At the provider and system level, provider restrictions may be associated with system inefficiencies that increase administrative burden, workload and staff time	Provider restrictions engage states’ obligation to respect, protect and fulfil rights to life and health (by ensuring abortion regulation is evidence-based and proportionate, and by ensuring that where it is lawful abortion is safe and accessible)	Provider restrictions are linked with system costs. Where these restrictions are not justified by evidence (e.g., of competence, effectiveness, acceptability) they interfere disproportionately with rights to health and to physical and mental integrity
Perceived imposition on personal ethics or conscience	Overall, evidence from one study suggests that provider restrictions by means of mandated counselling may have a perceived imposition on providers’ personal ethics or conscience	Provider restrictions engage states’ obligation to respect, protect and fulfil rights to life and health (by ensuring abortion regulation is evidence-based and proportionate, and by protecting healthcare professionals providing abortion care)	Provider restrictions that are not justified by evidence (e.g., of competence, effectiveness, acceptability) may interfere with the right of healthcare providers to thought, conscience or belief by prohibiting them conscientiously from providing abortion care and reducing or hindering access to lawful abortion
Perceived impact on relationship with patient	Overall, evidence from one study suggests that provider restrictions by means of mandated counselling are perceived by some providers to have a negative impact on the provider-patient relationship	Provider restrictions engage states’ obligation to respect, protect and fulfil rights to life and health (by ensuring abortion regulation is evidence-based and proportionate, and by protecting healthcare professionals providing abortion care)	Provider restrictions that are not justified by evidence (e.g., of competence, effectiveness, acceptability) interfere disproportionately with rights to health and to physical and mental integrity
Stigmatization	No evidence identified	Provider restrictions engage states’ obligation to respect, protect and fulfil rights to life and health (by protecting healthcare professionals providing abortion care)	Provider restrictions may intensify or exacerbate abortion-related stigma for healthcare providers permitted to provide abortion care. Stigma may result in decisions to opt out of or minimize abortion care provision, with consequences for the availability of lawful abortion

One study [20] provided evidence on the impact of the intervention on the outcomes, perceived *imposition on personal ethics or conscience,* and *perceived impact on the provider-patient relationship.* This study showed that where the law requires provision by a specific provider, who must also undertake mandated scripted counselling, the professionals perceive this to be an unreasonable intrusion into the practice of medicine and as having a negative impact on the provider-patient relationship. Thus, as well as arguably imposing on the health worker’s right to freedom of conscience or belief, such restrictions may reduce the quality of sexual and reproductive health care that pregnant people receive and thus be inconsistent with the right to the highest attainable standard of physical and mental health.

## Discussion

The evidence from this review suggests that provider restrictions have implications for access to quality abortion. The right to sexual and reproductive health obliges states to ensure that health-care facilities, goods and services are available, accessible, acceptable and of good quality ([4], paras 8, 12), which the evidence from this review suggests is undermined by provider restrictions. Furthermore, although there are some exceptions [24], the rate at which physicians and other healthcare providers tend to take up opportunities for abortion training where they are available is low [25], training in surgical abortion provision is not always a requirement of qualification [26], and there are often shortcomings in abortion training provided in obstetrics and gynaecology training contexts [27]. Given this, any regulatory approaches that may reduce the number of willing providers with foreseeable implications for the availability and accessibility of abortion require significant justification on the part of the state and raise questions of human rights compliance.

International human rights law requires states to take steps to ensure women do not have to undergo unsafe abortion ([28], para 10), to reduce maternal morbidity and mortality, and to effectively protect women and girls from the physical and mental risks associated with unsafe abortion ([6], para 8; 28, para 10). States must revise their laws to ensure this ([6]; 28, para 10; [29], para 44). In practice, this means that the regulation of abortion must not jeopardize women’s lives, subject women or girls to physical or mental pain or suffering constituting torture or to cruel, inhuman or degrading treatment or punishment, discriminate against women or girls, or interfere arbitrarily with their privacy ([6], para 8). Given the evidence presented in this review suggesting that provider restrictions contribute to delays and recourse to unsafe abortion, a human rights-based approach to abortion regulation would require the removal of overly restrictive provider restrictions. The review also provides evidence that speaks to possible routes for regulatory reform by expanding the health workforce involved in abortion related care, as well as expanding health workers roles, both of which could improve timely access to first trimester surgical and medical abortion, reduce costs, save time, and reduce the need for travel [19]. Among the WHO’s core functions are “shaping the research agenda and stimulating the generation, translation, and dissemination of valuable knowledge” and “setting norms and standards, and promoting and monitoring their implementation” [30]. Accordingly, the *Abortion Care Guideline* is “intended to provide concrete information and guidance…for national and subnational policy-makers, implementers and managers…members of nongovernmental organizations and other civil society organizations and professional societies…health workers and other stakeholders” ([11], p. 3) to support an enabling environment for quality abortion. Among the components of an enabling environment is respect for human rights including a supportive framework for law and policy ([11], p. 5) in which non-clinical barriers to abortion are removed. The *Guideline* accordingly recommends against regulation of who can provide and manage abortion (i.e. provider restriction) that is inconsistent with WHO guidance ([11], p. 59).

It is further important to note that in many settings provider restrictions interact with other abortion laws and policies, which may compound their effects. For example, where abortion is criminalized (i.e. where abortion is contained in penal codes or criminal laws, other offences are applied to abortion-related activity, or there are criminal penalties for having, assisting with, providing information about, or providing abortion) provider restrictions indicate boundaries of criminal liability. In other words, provision by a specified provider is lawful while provision by a non-specified provider is unlawful. The combined effect of these regulatory interventions (provider restrictions plus criminalization) can be to create ‘chilling effects’ for healthcare professionals who may be unwilling to engage in abortion care provision in case of incurring criminal liability. The negative human rights implications of criminalization are widely recognized by regional human rights courts and treaty monitoring bodies ([6], para 8; [8], paras 20, 34; 28; [31], para 18; [32], para 51(l); [33], para 60; [34], paras 79–83, 107; [28], para 20; [35]), and form part of the broader regulatory context in which interventions such as provider restrictions must be understood.

## Limitations

This review has limitations. It is limited in geographic scope, with papers relating to just four settings (Australia, Ethiopia, Nepal and United States of America (USA)), and in some cases only to the law in sub-national jurisdictions (the states of California (USA), Illinois (USA) and Victoria (Australia)). This review also only contains manuscripts published in English. Further research on the impact of provider restrictions in a wider range of settings would be welcome. Furthermore, the realization of human rights applicable to abortion-related interventions is not a research area that readily lends itself to randomized controlled trials or comparative observational studies; rather, studies are often conducted without comparisons. While this may be considered a limitation from a standard methodological perspective for systematic reviews, it does not limit the ability to identify human rights-related implications of law and policy interventions. Additionally, standard tools for assessing risk of bias or quality, including GRADE [36], were unsuitable for our review, given the objective of fully integrating human rights standards into our understanding of the effects of provider restrictions as a regulatory intervention. Thus, it was necessary to review and include a wide range of evidence from legal analyses to clinical studies. Finally, and consistent with the methodological approach pursued [9], this review applies international, rather than regional or domestic, human rights law to develop a general understanding of the rights-related implications of provider restrictions. The applicability of any individual human rights standard in a specific setting will depend on factors including the state’s ratification of relevant human rights instruments and their status in domestic law ([11], p. 7).

## Conclusion

This review identified evidence of the impacts of provider restrictions on people seeking to access abortion and on abortion providers. When considered alongside international human rights law, this evidence pointed clearly to impacts that have negative implications for health outcomes, health systems, and human rights. This is especially so as international guidance provided by the WHO indicates best practice in provision and management of abortion and shows clearly that undue provider restrictions are not justified by reference to the nature and complexity of abortion [2, 3, 11]. Given this, and as international human rights law enjoins evidence-based regulation, where they exist, provider restrictions should operate to maximize health outcomes, health system efficiency, and human rights enjoyment.

## Supplementary Information

**Additional file 1. **Search strategy.

**Additional file 2: Table S1.** Impact on the intervention on abortion seekers. **Table S2.** The impact of the intervention on health professionals

## Data Availability

All data generated or analysed during this study are included in this published article and its Additional files.

## Abbreviations

USA: United States of America
WHO: World Health Organization
