# Supplementary material for: The impact of provider restrictions on abortion-related outcomes: a synthesis of legal and health evidence
Source: Reprod Health. 2022 Apr 18;19:95. doi: 10.1186/s12978-022-01405-x (PMC9014563; doi:10.1186/s12978-022-01405-x)
Supplement: Supplementary file 1 — Additional file 1. Search strategy. [file 12978_2022_1405_MOESM1_ESM.docx]

**SEARCH STRATEGY**

**Limitations:**

- Year: from 2010
- Study designs: no restrictions, PhD thesis ok
- Language or geographics: no restrictions

**Databases:** PubMed, HeinOnline, JStor, Google Scholar (importing done through Publish or Perish)

All Databases

abortion AND provider restriction | abortion AND provider regulation | abortion AND nurse | abortion AND midwife | abortion AND midwives | abortion AND provider regulation | abortion provider restriction | abortion AND pharmacists | abortion AND “health professionals” | abortion AND “healthcare professionals” | abortion AND “healthcare providers”
