# Supplementary material for: The impact of provider restrictions on abortion-related outcomes: a synthesis of legal and health evidence
Source: Reprod Health. 2022 Apr 18;19:95. doi: 10.1186/s12978-022-01405-x (PMC9014563; doi:10.1186/s12978-022-01405-x)
Supplement: Supplementary file 2 — Additional file 2: Table S1. Impact on the intervention on abortion seekers. Table S2. The impact of the intervention on health professionals [file 12978_2022_1405_MOESM2_ESM.docx]

*Additional file 2: Table S1. Impact on the intervention on abortion seekers*

| **OUTCOME: DELAYED ABORTION** | | | |
| --- | --- | --- | --- |
| **Studies** | **Direction of the evidence** | **What does this mean?** | **Overall conclusion** |
| Battistelli 2018^1^ | ▲ | Involving nurse-practitioners, certified nurse-midwives and physician assistants may improve abortion access and timely management of post abortion care for individuals obtaining first trimester surgical abortions. | Overall, evidence from three studies suggests that provider restrictions may result in delayed abortions.  One study indirectly examines provider restrictions on delayed abortion by demonstrating how expansion of health worker roles (and thereby reducing provider restrictions) improve timely access to first trimester surgical and medical abortion.  Evidence from two studies suggests that government mandated abortion counselling increases the administrative and logistical burdens for providers and women, and may increase abortion delays. |
| Mercier 2015^1^ | ▲ | Government mandated scripted abortion counselling laws increase logistical and psychological burdens for abortion providers. Mandated counselling is provided in addition to, not as a replacement for, existing clinical standards and processes for informed consent. These laws may increase abortion delays for some women. |  |
| Srinivasulu 2021 | ▲ | Prohibiting APRNs and physician assistants from providing abortion care meant that abortion could only be provided when a physician travelled to the clinic twice a month to provide. |  |
| **OUTCOME: OPPORTUNITY COSTS** | | | |
| Afework 2015^1^ | ▲ | Involving health extension workers in abortion care may reduce costs and need to travel, as well as save time for abortion seekers. | Overall evidence from seven studies suggests that provider restrictions increase opportunity costs for abortion seekers.    Provider restrictions may be linked to opportunity costs such as increased financial costs, need for travel, waiting times, additional clinic contacts, emotional distress, and undesired surgical interventions. |
| Andersen 2016^1^ | ▲ | Training of auxiliary nurse-midwives increases access to abortion care in rural areas, and at the primary healthcare level. |  |
| Battistelli 2018^1^ | ▲ | Involving nurse-practitioners, certified nurse-midwives and physician assistants in abortion care means that a greater number of first trimester abortion procedures can be conducted and waiting times are reduced. |  |
| Grossman 2015 | ▲ | Expanding mifepristone prescription rights to pharmacists may increase access to medical abortion. |  |
| Mercier 2015^1^ | ▲ | Government mandated scripted counselling prior to an abortion is perceived by providers to be an obstacle for women seeking an abortion, and a cause of emotional distress. Government mandated counseling leads to an additional appointment needed for abortion care. |  |
| Rasmussen 2021 | ▲ | Prohibiting pharmacist provision of mifepristone for abortion was perceived to lead to increased stigmatization of abortion. |  |
| Srinivasulu 2021 | ▲ | Provider restrictions result in patients being referred to outside facilities who in turn experienced added wait times and logistical issues, leading to delays in care and disproportionate financial burdens. In some cases, women had unwanted and painful surgical procedures due to the lack mifepristone. |  |
| **OUTCOME: SELF-MANAGED ABORTION** | | | |
| Afework 2015^1^ | ▲ | Involving health extension workers in abortion care may prevent unsafe self-managed abortions.^2^ | Overall evidence from one study suggests that provider restrictions, when they limit access to care, may be linked to unsafe self-managed abortion |

▲ = the intervention leads to an increase in the outcome; ○ = the intervention leads to no change in the outcome; Ñ= the intervention leads to a decrease in the outcome. Symbol does not indicate magnitude or certainty of effect

^1^ Qualitative study design: tests of statistical significance not applicable

^2^ Concerns about adequacy exist – data underlying the finding is not sufficiently rich, data come from a small number of studies and few participants

*Additional file 2: Table S2.* The impact of the intervention on health professionals

| **OUTCOME: WORKLOAD IMPLICATIONS** | | | |
| --- | --- | --- | --- |
| **Studies** | **Direction of the evidence** | **What does this mean?** | **Overall conclusion** |
| Andersen 2016^1^ | ▲ | Involving auxiliary nurse-midwives in abortion care improves sustainability of services when auxiliary nurse-midwives are local to their communities. This leads to more consistent availability of abortion care at the primary healthcare level. | Overall evidence from six studies suggests that provider restrictions have workload implications.    Four of the five studies examined this indirectly, by demonstrating the benefit in task sharing abortion care with health workers who are not physicians. One study directly examined workload implications from provider restrictions with mandated counselling.    All studies reported that provider restrictions may be linked with a range of workload implications including issues surrounding sustainability of staffing, logistical and financial costs, organizational changes, increased workload and stress among providers. |
| Battistelli 2018^1^ | ▲ | Employing nurse-practitioners, certified nurse-midwives and physician assistants to provide surgical first trimester abortion care may require changes to staffing, logistical and financial costs to the organization in the short term. |  |
| Bridgman-Packer 2018^1^ | ▲ | Expanding health worker roles in abortion care shifts components of care away from physicians and expands access to care. |  |
| De Moel-Mandel 2019^1^ | ▲ | Expanding health worker roles in abortion care to midwives requires challenging the current traditional doctor nurse distribution of labor. Requiring physician involvement in prescriptions and for public finance reimbursement introduces inefficiencies even where nurses are permitted to provide abortion care. |  |
| Mercier 2015^1^ | ▲ | When government mandated scripted counselling is required by a healthcare professional, it requires changes to bureaucracy and staffing as well as changes to modes of counselling. The process and legal obligation to ensure compliance, and coordinating necessary logistics, increases the psychological workload of abortion providers. |  |
| Rasmussen 2021 | ▲ | Prohibiting pharmacist provision of mifepristone for abortion prevents providers from undertaking their full scope of practice and poses difficulties in addressing implementation barriers including unsupportive peers, stocking mifepristone, and disruptions in clinic flow. |  |
| **OUTCOME: SYSTEM COSTS** | | | |
| Battistelli 2018^1^ | ▲ | Involving nurse-practitioners, certified nurse-midwives and physician assistants in first-trimester abortion care may reduce system costs in several ways including by means of reduced staff expenses in settings of low reimbursement for abortion care, by averting costs from unwanted births, and delayed management of complications. | Overall, evidence from five papers suggests that provider restrictions contribute to increased system costs.    Provider restrictions contribute to costs at the individual, provider and systems level. For individuals these costs are typically associated with increased time in obtaining care. At the provider and system level, provider restrictions may be associated with system inefficiencies that increase administrative burden, workload and staff time. |
| De Moel-Mandel 2019^1^ | ▲ | Health system reimbursement processes and requirements result in system costs and inefficiencies, even when medication abortion is nurse-led. For example, provider restrictions may involve funding models and regulatory structures that require physician involvement for billing, reimbursement and prescriptions. |  |
| Mercier 2015^1^ | ▲ | Government mandated scripted counselling by a healthcare professional prior to an abortion, incurs increased institutional costs due to staff time. |  |
| Rasmussen 2021 | ▲ | Prohibiting pharmacist provision of mifepristone for abortion led to uncertainty about prescription of mifepristone for management of early pregnancy loss. |  |
| Srinivasulu 2021 | ▲ | Provider restrictions result in the provision of unwanted aspiration because of a lack of mifepristone in clinics. This arose for women experiencing early pregnancy loss as well as those seeking abortion. |  |
| OUTCOME: PERCEIVED IMPOSITION ON PERSONAL ETHICS OR CONSCIENCE | | | |
| Mercier 2015^1^ | ▲ | ^2^When government mandated scripted counselling is required by a healthcare professional, some abortion providers perceive this as an unreasonable intrusion into the practice of medicine. | Overall, evidence from one study suggests that provider restrictions by means of mandated counselling may have a perceived imposition on providers’ personal ethics or conscience. |
| OUTCOME: PERCEIVED IMPACT ON RELATIONSHIP WITH PATIENT | | | |
| Mercier 2015^1^ | ▲ | ^2^When government mandated scripted counselling is required by a healthcare professional, abortion providers consider it as having a negative impact on the provider-patient relationship. | Overall, evidence from one study suggests that provider restrictions by means of mandated counselling are perceived by some providers to have a negative impact on the provider-patient relationship. |

▲ = the intervention leads to an increase in the outcome; ○ = the intervention leads to no change in the outcome; Ñ= the intervention leads to a decrease in the outcome. Symbol does not indicate magnitude or certainty of effect

^1^ Qualitative study design: tests of statistical significance not applicable
